# Goiter in the paintings by Rogier van der Weyden (1399–1464)

**DOI:** 10.1007/s40618-023-02108-1

**Published:** 2023-05-12

**Authors:** H. Ashrafian

**Affiliations:** The Department of Surgery and Cancer, Institute of Global Health Innovation, Imperial College London, St Mary’s Hospital, 10th Floor Queen Elizabeth the Queen Mother (QEQM) Building, Praed Street, London, W2 1NY UK

**Keywords:** Thyroid, Goiter, Iodine deficiency

Rogier van der Weyden (1399–1464) is regarded as one of the forefathers of Northern Renaissance art. Born in Tournai, Belgium in 1399/1400 he became a painter notably influenced by Jan van Eyck [1], and rapidly demonstrated his own innovation, for example presenting a character (Saint Luke most likely as a self-portrait) directly in the presence of the Virgin character, a novel stylistic revolution for that time.

I now note examples of Goiters in several paintings by Rogier van der Weyden (Fig. 1a–d.). While other examples do exist in Northwestern Europe and Southern Europe, where goiter is presented through fullness and the signs of da Vinci Sign (loss or shallowing of the suprasternal notch recess) or Botticelli (cranio-cervical neck flexion accentuating thyroid enlargement) [2], the cases presented by van der Weyden are among the earliest.

**Fig. 1:**
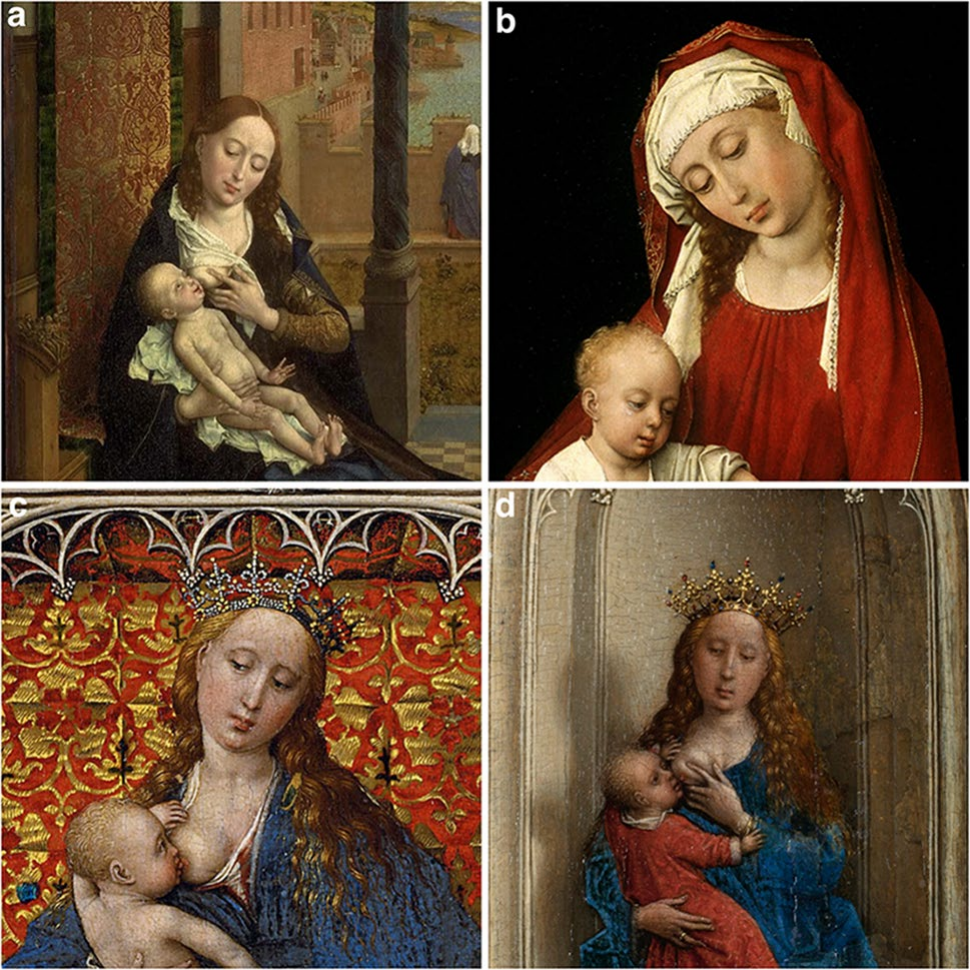
Rogier van der Weyden **a** Saint Luke Drawing the Virgin (1435–40) © Boston Museum of Fine Arts, Boston, USA **b** The Durán Madonna (c.1435–38) © Museo del Prado, Madrid, Spain **c** The Madonna Standing (c.1430–1432) © Kunsthistorisches Museum, Vienna, Austria **d** The Virgin and Child Enthroned (1433) © Thyssen-Bornemisza Museum, Madrid, Spain

The goiters presented (concomitant with the stylism of icon paintings in the eyelids and skin color hues), could have been representative of realistic underlying pathology that may have originated from endemic iodine deficiency (rather than autoimmunity and orbitopathy seen in other Renaissance paintings) [3, 4]. This early Northern Renaissance work highlights the pertinence of thyroid disease in across the breadth of Renaissance Europe presented through the hands of genius master artists of the era.

## References

[1] Ashrafian H (2018). Hypothyroidism in the “Arnolfini Portrait” (1434) by Jan Van Eyck (1390–1441). J Endocrinol Invest.

[2] Ashrafian H (2023). Goiters in the renaissance era multiple cases of thyroid autoimmunity and iodine deficiency. Best Pract Res Clin Endocrinol Metab.

[3] Ashrafian H (2023). Differential diagnosis of a Thyroid mass, Gottron’s papules, Calcinosis Cutis and Ptosis on the Saint Mary Magdalen and two depictions of a madonna and baby by Bartolomeo Vivarini (1432–1499). J Endocrinol Invest.

[4] Trimarchi F, Martino E (2023). Endemic goiter in two masterpieces by Piero della Francesca (c.1415–1492). J Endocrinol Invest.

